# Resuscitative strategies in traumatic hemorrhagic shock

**DOI:** 10.1186/2110-5820-3-1

**Published:** 2013-01-12

**Authors:** Adrien Bouglé, Anatole Harrois, Jacques Duranteau

**Affiliations:** 1 Departement of Anesthesia and Intensive Care, Bicêtre Hospital, Hôpitaux universitaires Paris-Sud, Université Paris-Sud, Assistance Publique-Hôpitaux de Paris, 78, rue du Général Leclerc, 94275, Le Kremlin Bicêtre, France; 2 Medical Intensive Care Unit, Cochin Hospital, Groupe Hospitalier Cochin Broca Hôtel-Dieu, Assistance Publique des Hôpitaux de Paris, 27, rue du Faubourg Saint-Jacques, 75014, Paris, France

**Keywords:** Trauma, Hemorrhagic shock, Fluid resuscitation, Vasopressors, Acute coagulopathy of trauma

## Abstract

Managing trauma patients with hemorrhagic shock is complex and difficult. Despite our knowledge of the pathophysiology of hemorrhagic shock in trauma patients that we have accumulated during recent decades, the mortality rate of these patients remains high. In the acute phase of hemorrhage, the therapeutic priority is to stop the bleeding as quickly as possible. As long as this bleeding is uncontrolled, the physician must maintain oxygen delivery to limit tissue hypoxia, inflammation, and organ dysfunction. This process involves fluid resuscitation, the use of vasopressors, and blood transfusion to prevent or correct acute coagulopathy of trauma. The optimal resuscitative strategy is controversial. To move forward, we need to establish optimal therapeutic approaches with clear objectives for fluid resuscitation, blood pressure, and hemoglobin levels to guide resuscitation and limit the risk of fluid overload and transfusion.

## Review

### Introduction

Hemorrhage remains the major cause of preventable death after trauma [1]. In the acute phase of hemorrhage, the physician’s therapeutic priority is to stop the bleeding as quickly as possible. Hemorrhagic shock is a pathologic state in which intravascular volume and oxygen delivery are impaired. As long as this bleeding is not controlled, the physician must maintain oxygen delivery to limit tissue hypoxia, inflammation, and organ dysfunction. This procedure involves fluid resuscitation, use of vasopressors, and blood transfusion to prevent or correct traumatic coagulopathy. However, the optimal resuscitative strategy is controversial: choice of fluid for resuscitation, the target of hemodynamic goals for hemorrhage control, and the optimal prevention of traumatic coagulopathy are questions that remain. This review focuses on new insights into resuscitative strategies in traumatic hemorrhagic shock.

### Fluid resuscitation

Fluid resuscitation is the first therapeutic intervention in traumatic hemorrhagic shock. We discuss the choice of the type of fluid for resuscitation. There is no proof in the literature that supports the superiority of one type of fluid over another type of fluid in trauma patients. The most important dual advantage that colloids have over crystalloids is that colloids can induce a more rapid and persistent plasma expansion because of a larger increase in oncotic pressure, and they can quickly achieve circulatory goals. Although crystalloids are cheaper, research findings have shown no survival benefit when colloids are administered. However, resuscitation with large volumes of crystalloids has been associated with tissue edema, an increased incidence of abdominal compartment syndrome [2], and hyperchloremic metabolic acidosis [3].

The SAFE study demonstrated that albumin administration was safe for fluid resuscitation for intensive care unit (ICU) patients and that there was no difference in the mortality rate of patients who were treated with albumin and saline [4]. In a subgroup of trauma patients, the investigators observed a positive trend in benefit for saline use over albumin use. This difference in the relative risk of death was due to the greater number of patients, who had trauma and an associated brain injury and who died after random assignment to the albumin-treated group as opposed to the saline-treated group. No mechanism was offered to account for this finding, but the low hypo-osmolarity of albumin may increase the risk of brain edema. A recent Cochrane review [5] in critically ill patients (patients with trauma, burns, or after surgery) reported no evidence accumulated from RCTs that resuscitation with colloids reduced the risk of death compared with resuscitation with crystalloids. In a review of clinical studies dating to 2002 with safety data documented in ICU patients who received HES, gelatin, dextran, or albumin, Groeneveld et al. [6] demonstrated that impaired coagulation, clinical bleeding, and acute kidney injury (AKI) were frequently reported after HES infusion. Notably, this analysis was strongly influenced by the VISEP study (Volume Substitution and Insulin Therapy in Severe Sepsis study) [7], in which a former-generation HES was used (200/0.5) with doses that exceeded the recommended maximal doses. These meta-analyses take into account heterogeneous populations of patients with different therapeutic strategies. Recently, Perner et al. [8] have shown an increased risk of death (dead on day 90) in patients with severe sepsis who were assigned to receive fluid resuscitation with HES 130/0.42 (6% HES 130/0.42 in Ringer’s acetate, last generation of HES) compared with those who received Ringer’s acetate. Moreover, more patients required renal-replacement therapy in the HES 130/0.42 group (22%) than in the Ringer’s acetate group (16%). In light of the shared pathophysiological pathways with inflammation activation between sepsis and trauma, the use of HES raises serious concerns with respect to its safety in trauma patients [9].

Thus, there is an imperative need to study trauma patients who are in hemorrhagic shock. Recently, a double-blind, randomized, controlled study that compared 0.9% saline vs. hydroxyethyl starch (HES 130/0.4) was conducted in penetrating blunt trauma patients who required >3 liters of fluid resuscitation [10]. In patients with penetrating trauma (n = 67), the use of HES (130/0.4) was associated with a better lactate clearance, thus suggesting early resuscitation. Furthermore, lower maximum SOFA scores and an absence of acute renal injury were observed in the HES group. However, in patients with blunt trauma (n = 42), there was no difference in fluid requirements, lactate clearance, and maximum SOFA scores between the two groups. In addition, a greater requirement for blood and blood products was reported in the HES group with a significantly greater alteration in coagulation (thromboelastography). It is difficult to draw conclusions, because patients in the HES group were more severely injured than patients in the saline group; we should apply caution when we interpret the results, because the study is based on a small sample size.

The last European guidelines for the management of bleeding after severe injury [11] recommended that crystalloids should be applied initially to treat the bleeding trauma patients and that the addition of colloids should be considered in hemodynamically unstable patients. Among colloids, HES or gelatin solutions should be used. The guidelines recommended using the new-generation HES within the prescribed limits because of the risks of AKI and alteration in coagulation.

Hypertonic saline (HTS) is an interesting tool in traumatic hemorrhagic shock. HTS has the major benefit of rapidly expanding blood volume with the administration of a small volume, especially if it is used with a colloid. Furthermore, HTS can be used as a hyperosmolar agent in patients with elevated intracranial pressure. However, HTS failed to improve outcomes in recent RCTs [12,13]. Bulger et al. [12] reported that HTS + dextran out-of-hospital resuscitation did not decrease survival without acute respiratory distress syndrome at 28 days in a blunt trauma population with a prehospital systolic blood pressure (SAP) ≤ 90 mmHg. However, benefit was observed in the subgroup of patients who required 10 U or more of packed red blood cells in the first 24 h. Recently, the same authors were unable to demonstrate an improvement in survival as a result of out-of-hospital administration of SSH + dextran in patients in hemorrhagic shock (SAP ≤ 70 mmHg or SAP 71–90 mmHg with heart rate ≥ 108 bpm) [13]. Moreover, a higher mortality rate was observed in patients who received HTS in the subgroup of patients who did not receive any blood transfusions in the first 24 hr. To explain this effect, the authors hypothesized that the out-of-hospital administration of SSH could mask the signs of hypovolemia and delay the diagnosis of hemorrhagic shock. Finally, the out-of-hospital administration of SSH to patients with severe traumatic brain injury did not improve their neurological function recovery.

### Vasoactive agents

Fluid resuscitation is the first strategy to restore mean arterial pressure in hemorrhagic shock. However, vasopressor agents also may be transiently required to sustain life and maintain tissue perfusion in the presence of a persistent hypotension, even when fluid expansion is in progress and hypovolemia has not yet been corrected. This point is crucial, because tissue perfusion is directly related to the driving pressure (the difference between pressures at the sites of entry and exit of the capillary), the radius of the vessel, and the density of capillaries; additionally, tissue perfusion is inversely related to blood viscosity. Thus, arterial pressure is a major determinant of tissue perfusion.

Norepinephrine (NE), which often is used to restore arterial pressure in septic and hemorrhagic shock, is now the recommended agent of choice during septic shock [14]. NE is a sympathomimetic agent with predominantly vasoconstrictive effects. NE exerts both arterial and venous α-adrenergic stimulation [15]. In addition to its arterial vasoconstrictor effect, NE induces venoconstriction (especially at the level of splanchnic circulation), which induces an increase in pressure in the capacitance vessels and actively shifts the venous blood volume to the systemic circulation [16]. This venous adrenergic stimulation may recruit blood from the venous unstressed volume, i.e., the blood volume that fills the blood vessels without generating an intravascular pressure. Moreover, stimulation of β_2_-adrenergic receptors decreases venous resistance and increases venous return [16]. Poloujadoff et al. [17], in an animal study during uncontrolled hemorrhage, suggested that NE infusion reduced the amount of fluid required to achieve a given arterial pressure target and corresponded to lower blood loss and significantly improved survival. We can therefore propose the early use of NE to restore blood pressure as quickly as possible and limit fluid resuscitation and hemodilution. However, the effects of NE have not been rigorously investigated in humans who suffered traumatic hemorrhagic shock. An analysis performed during a multicenter, prospective, cohort study designed to evaluate the outcome of adults who suffered blunt injury and who were in hemorrhagic shock proposed that the early use of vasopressors for hemodynamic support after hemorrhagic shock may be deleterious, compared with the aggressive use of volume resuscitation, and should be approached cautiously [18].

**Figure 1:**
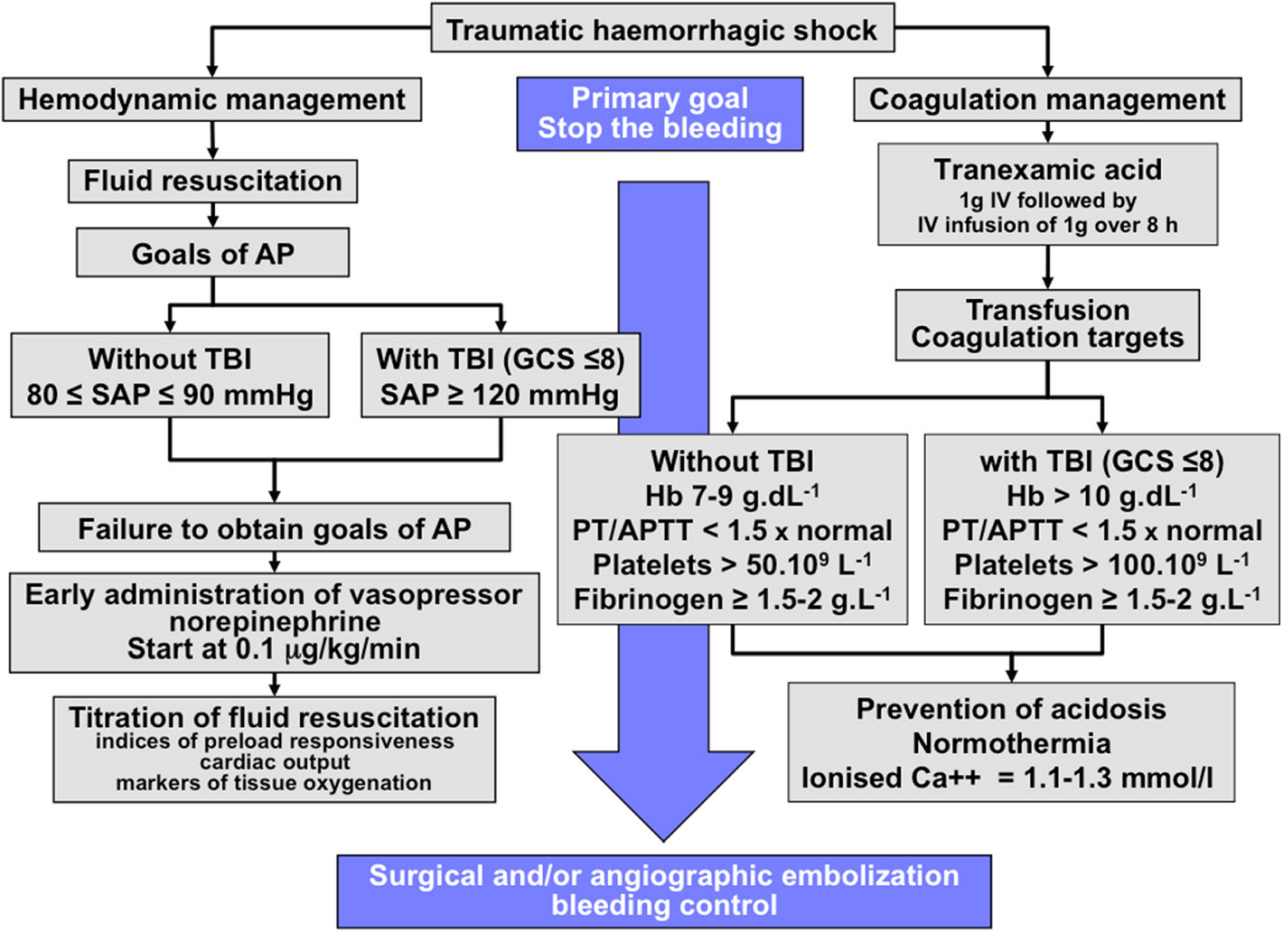
**Flowchart of initial management of traumatic hemorrhagic shock.** In the acute phase of traumatic hemorrhagic shock, the therapeutic priority is to stop the bleeding. As long as this bleeding is not controlled, the physician must manage fluid resuscitation, vasopressors, and blood transfusion to prevent or treat acute coagulopathy of trauma. AP, arterial pressure; SAP, systolic arterial pressure; TBI, trauma brain injury; Hb, hemoglobin; PT, prothrombin time; APTT, activated partial thromboplastin time.

This study has several limitations. First, this was a secondary analysis of a prospective, cohort study and was not designed to answer the specific hypothesis tested; second, the group that received vasopressors had a higher incidence of thoracotomy. Thus, a prospective study to define the effect of vasopressors used in patients with hemorrhagic shock is required. In conclusion, vasopressors may be useful if they are used transiently to sustain arterial pressure and maintain tissue perfusion during persistent hypotension, despite fluid resuscitation (Figure 1). Moreover, the early use of NE could limit fluid resuscitation and hemodilution. If we use NE at an early stage, we must note the recommended objectives of arterial pressure (SAP 80–100 mmHg) [11]. Thus, the dose of NE should be titrated until we reach the target SAP (Figure 1). Then, fluid resuscitation should be pursued and titrated according to indicators of preload responsiveness, cardiac output, and tissue oxygenation markers.

Because vasopressors may increase cardiac afterload when there is excessive infusion rate or impaired left ventricular function, it is essential to assess cardiac function during the initial ultrasound examination. Cardiac dysfunction may be altered in the trauma patient after cardiac contusion, pericardial effusion, or secondary to brain injury with intracranial hypertension. The presence of myocardial dysfunction requires treatment with an inotropic agent, such as dobutamine or epinephrine. In the absence of an evaluation of cardiac function or cardiac output monitoring, which often is observed in patients in the acute phase of hemorrhagic shock, we should suspect cardiac dysfunction in the presence of a poor response to fluid expansion and NE.

### Which objectives of fluid resuscitation and blood pressure?

The mean arterial pressure, which represents the perfusion pressure of all organs (except the heart), might serve as a target that physicians must achieve by early fluid administration. A critical element of the resuscitation of the patient with hemorrhagic shock is to prevent a potential increase in bleeding by a resuscitative manoeuvre that is overly aggressive. Fluid resuscitation may promote coagulopathy by diluting coagulation factors and favoring hypothermia. Moreover, an excessive level of mean arterial pressure (MAP) can favor the bleeding by preventing clot formation. Two concepts have emerged in past years: the concept of “low-volume resuscitation” and the concept of “hypotensive resuscitation.” Often, these two concepts are merged. Several experimental studies have suggested that the limited administration of fluids associated with a low blood pressure level as an end point may limit bleeding without the related increased risk of death [19]. Bickell et al. [20] in 1994 tested this concept in hypotensive patients with penetrating injuries to the torso. They compared immediate and delayed fluid resuscitation and reported that aggressive administration of intravenous fluids should be delayed until the time of operative intervention. Thus, Bickell et al. supported the concept of bringing the patient as quickly as possible to the trauma center and restricting fluid resuscitation until the time of operative intervention. Recently, a retrospective cohort study of patients from the American Trauma Data Bank [21] suggested that there was no survival benefit for prehospital IV placement or IV fluid administration. This concept could be limited by factors, such as older patients, severe brain injuries, or longer prehospital transport times (rural trauma). Future studies are required to clarify the volume and the timing of fluid resuscitation before surgical or angiographic embolization bleeding control. Minimal volume resuscitation is preferable to aggressive volume resuscitation before active bleeding has been controlled. It is critical to prevent hemodilution by limiting fluid resuscitation and using an aggressive transfusion strategy. Additionally, despite adequate fluid resuscitation, only blood transfusion can improve tissue oxygenation [22]. Thus, one key message is that we must consider blood transfusion early during the management of hemorrhagic shock to improve microvascular oxygen delivery.

The optimal level of blood pressure during the resuscitation of the hemorrhagic shock patient is still debated. The initial objectives are to control the bleeding as soon as possible and to maintain a minimal arterial pressure to limit tissue hypoxia. Restoration of arterial pressure with uncontrolled bleeding exposes the patient to the risk of increased bleeding or of prevented clot formation. Dutton et al. [23] found that titrating the initial fluid therapy to a lower-than-normal systolic blood pressure (70 mmHg) during active hemorrhage did not affect the mortality rate. The low number and the heterogeneity of studied patients limit the conclusions of this study. For example, the average systolic blood pressure was equal to 100 ± 17 mmHg in the 70-mmHg group, because the blood pressure had increased spontaneously toward normal in some patients. Recently, Morrison et al. [24], while evaluating patients in hemorrhagic shock who required emergent surgery, compared an intraoperative, hypotensive, resuscitative strategy in which the target MAP was 50 mmHg with a standard fluid resuscitative strategy in which the target MAP was 65 mmHg. The hypotensive, resuscitative strategy was a safe strategy that resulted in a significant reduction in blood product transfusions and overall IV fluid administration with a decrease in postoperative coagulopathy. However, in this study, there was no MAP difference between the two groups (64.4 mmHg vs. 68.5 mmHg) despite the different MAP objectives. The authors attributed this absence of a MAP difference to faster control of the bleeding in the 50-mmHg group by inducing a spontaneous MAP increase in this group. Thus, there is an insufficient quality or quantity of evidence to determine an optimal blood pressure level during active hemorrhagic shock. However, European guidelines for the management of bleeding trauma patients recommended a target systolic blood pressure of 80 to 100 mmHg until major bleeding has been stopped in the initial phase after trauma for patients without brain injury [11] (Figure 1). When traumatic hemorrhagic shock is associated with severe brain injury, cerebral perfusion pressure must be maintained by increasing the arterial pressure to prevent secondary brain injury. Before monitoring the intracranial pressure, we must define the optimal level of arterial pressure by using transcranial Doppler to determine the best balance between an optimal cerebral perfusion and the risk of increased bleeding (Figure 1).

### Transfusion and prevention of acute coagulopathy of trauma

**Figure 2:**
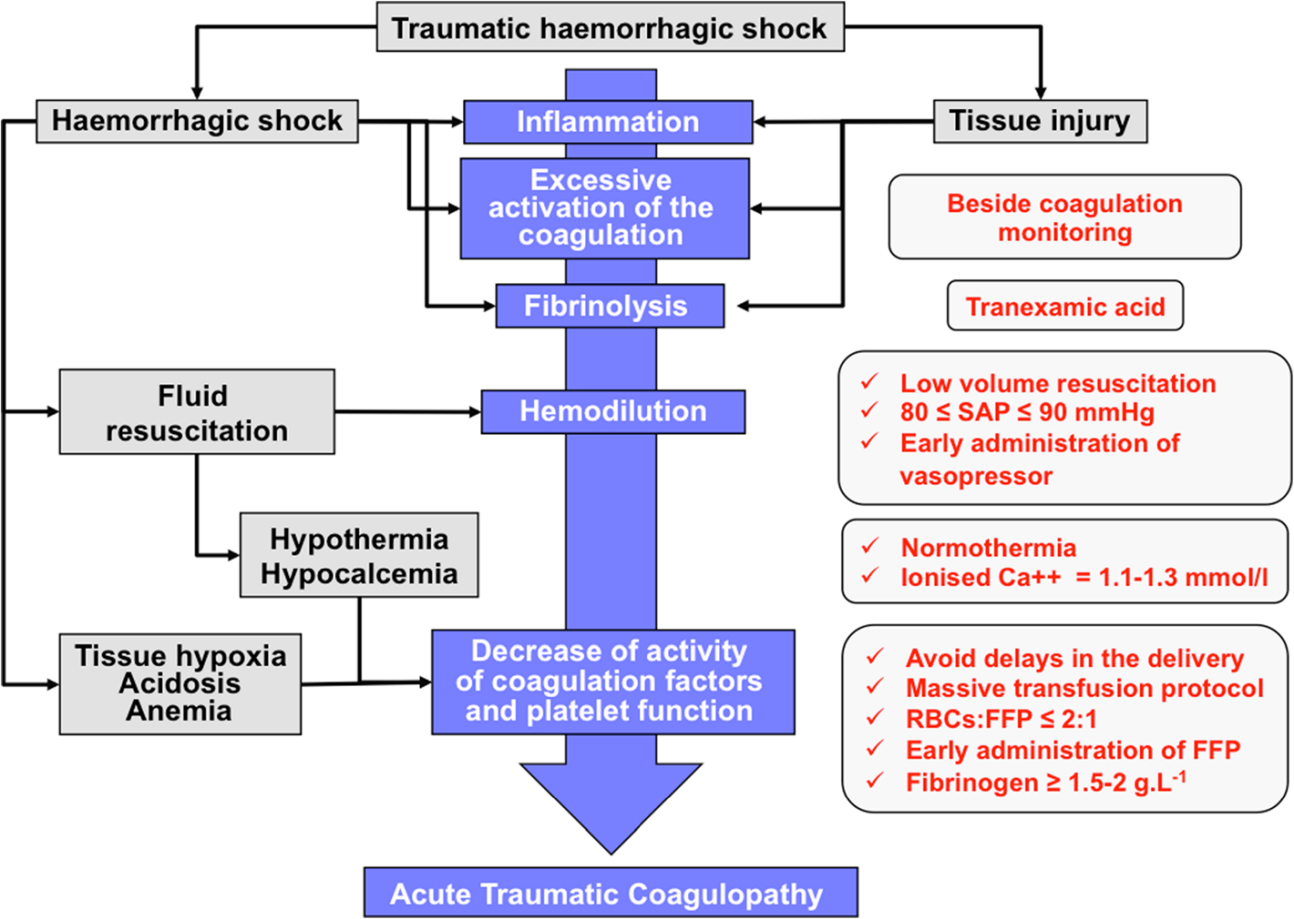
**The main pathophysiological mechanisms involved in acute traumatic coagulopathy and transfusion strategy.** SAP, systolic arterial pressure; RBC, red blood cells; FFP, fresh-frozen plasma.

The correction and prevention of traumatic coagulopathy (acute coagulopathy of trauma, ACoT) have become central goals of early resuscitative management of hemorrhagic shock. As Figure 2 illustrates, several interacting mechanisms contribute to the development of traumatic coagulopathy:

1) “Loss-dilution” phenomenon: bleeding and hemodilution secondary to fluid resuscitation cause a loss of coagulation factors and platelets.

2) Excessive activation of coagulation: the adapted activation of coagulation in response to hemorrhagic injury can become excessive under the influence of local or general phenomena. For example, tissue injury can cause endothelial injuries associated with local and systematic inflammatory reactions; these reactions are important for the production of tissue factor and factor VII, which can excessively activate coagulation.

3) Fibrinolysis: with an excessive activation of coagulation, a fibrinolytic response can overtake its physiological role of controlling coagulation.

4) Hypothermia: hypothermia favors alterations of platelet functions, coagulation factors, and fibrinolysis. Hypothermia is favored by an aggressive fluid resuscitation.

5) Acidosis: metabolic acidosis favors coagulopathy by means of a decrease in the activity of coagulation factors and platelet function and the degradation of fibrinogen.

6) Hypocalcemia: hemodilution induced by fluid resuscitation and citrate contained in blood products after massive transfusion contribute to hypocalcaemia.

7) Anemia: red blood cells have an important haemostatic role. The RBC flows maintain platelets close to the endothelial cells, and they can activate the platelet functions.

The risk of coagulopathy depends on the context. When bleeding occurs during surgery, the surgeon must immediately control the hemorrhage with a rapid fluid administration and RBC restoration to avoid or limit coagulopathy to only the “loss-dilution” phenomenon. However, in traumatic hemorrhagic shock, coagulopathy is frequent (from 10% to 34% of the trauma patients) and multifactorial [25,26], depending on the severity of the shock and trauma, and it is an independent factor of the morbidity and mortality in trauma patients.

It is crucial to avoid delays in the delivery of blood and blood components. Optimal hemostatic resuscitation requires prompt action with good communication and coordination between the treating clinicians and the transfusion service provider. Two major points in the management of these patients are: 1) regular assessment of the efficacy of replacement therapy using clinical assessment and monitoring of coagulation parameters, and 2) the use of an appropriate transfusion protocol with guidelines for its proper implementation.

Because there may be an unavoidable delay in processing and receiving laboratory results, more facilities are using point-of-care testing, which includes thromboelastography. Bedsides coagulation, monitoring in trauma patients by means of thrombelastography (TEG) or thromboelastometry (ROTEM) or activated clotting time (ACT) leads to an earlier and faster diagnosis of ACoT. Moreover, these monitoring devices enable personalized coagulation management, which serves to guide the coagulation therapy according to the real needs of the patient. We have observed that some clinical teams have changed their transfusion practices with goal-directed coagulation management based on TEG results [27,28].

Given the inherent delays involved with laboratory-guided transfusions and resuscitations, an institution that takes care of patients with massive hemorrhage must implement appropriate transfusion protocols and track blood product distribution. Establishing such protocols reduces the distribution and administration times of blood components. The fluidity of the prescription and distribution tracks of blood components might help to reduce the mortality rate for trauma patients who require a massive transfusion.

#### Red blood cells and fresh frozen plasma transfusion

The early administration of red blood cells (RBC) and fresh frozen plasma (FFP) is a priority to maintain arterial oxygen delivery and restore an effective coagulation. It is not possible to determine the optimal hemoglobin levels in patients with traumatic hemorrhagic shock, because no studies have assessed the relationship between hemoglobin levels and the adverse outcomes in patients with critical bleeding. In addition, the hemoglobin level target may depend on the patient’s medical history (age, history of cardiovascular diseases) and the type of trauma (presence or absence of brain injury). The administration of RBC is considered indispensable when the hemoglobin level is <7 g/dL [11] (Figure 1). This recommendation is based mainly on the results of the Transfusion Requirements in Critical Care (TRICC) study [29]. In this trial, Hebert et al. randomized hemodynamically stable, critically ill patients to either a liberal transfusion strategy, with target hemoglobin levels of 10–12 g/dL, or a restrictive strategy, with target hemoglobin levels of 7–9 g/dL. The mortality rate was similar in the two arms of the study, which indicated that a restrictive transfusion strategy was at least as safe as a liberal approach. In brain-injured patients, there are insufficient data to support restrictive or liberal hemoglobin levels [30,31]. However, many centers transfuse these patients to obtain a hemoglobin level of 10 g/dL. This strategy is based on the finding that an increased hemoglobin from 8.7 to 10.2 g/dL improved local cerebral oxygenation [32].

In the case of a major life-threatening hemorrhage, a patient could be transfused with O Rh-negative RBC units. Nevertheless, this practice must be considered the exception, and it must be implemented as part of a massive transfusion protocol.

The administration of FFP should be associated as soon as possible with RBC transfusion to compensate for the deficit in coagulation factors. The initial recommended dose is 10 to 15 ml/kg [11]. Additional doses will depend on the results of monitoring the coagulation parameters. FFP is recommended when PT or APTT is 1.5 times the normal value (Figure 1).

Several recent studies involving military or civil trauma patients have suggested the importance of an RBC/FFP ratio of approximately 1:1. However, these results should be interpreted carefully because of the potential for survival bias (that is, patients who die early are more likely to have received a higher RBC/FFP ratio). Thus, the optimal value of the RBC:FFP ratio remains controversial. Kashuk et al. [33] reported in civilian patients that a high RBC:FFP ratio (average 2:1) was associated with a better survival rate than a low RBC:FFP ratio (average 4:1), but these authors described a U-shaped relationship between the mortality risk and the RBC:FFP ratio with a critical threshold for survival in the range of 2:1 and 3:1 RBC:FFP. Thus, there is no absolute agreement on the optimal target RBC:FFP ratio. Additional research should be directed at defining this optimal target RBC:FFP ratio and identifying those patients who may benefit. The Australian and New Zealand guidelines on patient blood management suggested a ratio of ≤2:1:1 of RBC:FFP:platelets [34]. A similar recommendation has been recently established by the French Health Products Safety Agency (Agence nationale de sécurité du médicament et des produits de santé-AFSSAPS). The RBC:FFP ratio is an important element of the aggressive RBC and plasma resuscitation, but the time course for transfusion is a major element, and, more important than the crude RBC:FFP ratio, the early use of RBCs and FFP could improve the outcome of patients with traumatic hemorrhagic shock [35]. Therefore, it is critical to begin the plasma transfusion as quickly as possible (ideally at the same time as the RBC transfusion) (Figure 2). The essential concept is to have an aggressive plan to restore the biological hemostasis as quickly as possible to rapidly control the bleeding.

Early monitoring of coagulation is essential to identify coagulopathy during trauma and to facilitate goal-directed transfusion. However, conventional plasma-based coagulation tests, such as prothrombin time (PT), activated partial thromboplastin time (APTT), international normalized ratio (INR), fibrinogen, and platelet number, only reflect the initiation of the hemostatic process; the tests cannot be used to evaluate the amplification of propagation or increased fibrinolysis. Whole blood assays, such as TEG or ROTEM, provide rapid evaluation of clot formation, strength, and lysis, which reflect the entire hemostatic process [36,37]. There is emerging evidence for the clinical application of these bedside techniques during trauma. The use of these techniques has profoundly modified the transfusion strategy of some clinical teams. For instance, Schöchl et al. [27,28] explored goal-directed coagulation management using fibrinogen concentrate and prothrombin complex concentrate (PCC), administered according to ROTEM measurements. In a retrospective analysis, these authors compared patients from their trauma center and patients from a trauma register and reported that this goal-directed coagulation management strategy could reduce the need for RBC or platelet concentrate transfusion, in relation to FFP-based hemostatic therapy. RBC transfusion was avoided in 29% of the patients in the fibrinogen-PCC group compared with only 3% of the patients in the FFP group; there was a comparable mortality rate in both groups. This approach is interesting, especially with respect to the potential risks of transfusion. The transfusions of FFP and platelet concentrates have been associated with an increased risk of multiple organ dysfunction syndrome and acute respiratory distress syndrome [38-40]. However, the issue of an increased risk of venous thromboembolism with a fibrinogen concentrate-PCC strategy has not been addressed.

#### Platelet transfusion and fibrinogen concentrate

Platelet transfusion is recommended when platelets counts are <50.10^9^ L^-1^ (Figure 1). The platelet count should be maintained at a higher level in case of traumatic brain injury, i.e., 100.10^9^ L^-1^.

Fibrinogen is a mandatory compound in the coagulation pathway, and the plasma fibrinogen level should be corrected to anticipate clotting. The threshold for treatment with a fibrinogen concentrate or cryoprecipitate during acute bleeding was recently upgraded to a fibrinogen plasma level of less than 1.5 to 2.0 g/L (Figure 1). This new threshold is based on experimental and clinical TEG data, where fibrinogen administration during the acute phase of hemorrhagic shock was able to correct the TEG abnormalities. Unfortunately, the use of FFP failed to rapidly correct the hypofibrinogenemia secondary to bleeding. For example, Chowdary et al. [27] reported that resuscitation with 10 to 15 mL.kg^-1^ of FFP only increased the fibrinogen plasma level to 0.4 gL^-1^. More than 30 mL.kg^-1^ of FPP should be necessary to increase the fibrinogen plasma level to 1 g.L^-1^.

#### Tranexamic acid

Recently, a randomized, controlled trial that included 20,211 trauma patients [28] showed that the routine administration of tranexamic acid (loading dose of 1 g over 10 min, then infusion of 1 g over 8 hr) in patients with hemorrhagic shock was associated with a decreased mortality rate without an increase of thromboembolic complications. Thus, tranexamic acid should be included in the current management of patients with traumatic hemorrhagic shock (Figures 1 and 2). The optimal effect of this drug is observed in the first 3 hr of use [28].

#### Factor VIIa

Given the failure of recombinant Factor VIIa to decrease the mortality rate of patients in hemorrhagic shock [41], the use of this factor should be discussed on a case-by-case basis when the hemorrhagic shock cannot be controlled by surgical and/or angiographic hemostasis, and when the different biological parameters of hemostasis (i.e., hematocrit, platelets, PT, APTT, calcemia, and pH) are adequately corrected [42]. It is essential to balance its use with the real risk of thromboembolic events.

### Adjuvant therapeutics of hemorrhagic shock

Traumatic hemorrhagic shock is associated with an intense systemic inflammatory response. During the past decade, many therapeutic strategies were tested in the treatment of hemorrhagic shock, such as recombinant human activated protein C (APC), IL-1 receptor antagonist, anti-TNF or anti-LPS agents, or tight glycemia control. However, these treatments were ultimately ineffective and sometimes harmful.

Recently, a multicenter trial demonstrated that the administration of hydrocortisone in trauma patients was associated with a significantly reduced risk of developing pneumonia (36% vs. 51%) and a decrease in the duration of mechanical ventilation [30]. No difference in the mortality rate was observed between the two groups. We should, however, be cautious before recommending the early use of corticosteroids after trauma. The CRASH study, which investigated the use of corticosteroids after severe traumatic brain injury in more than 10,000 patients, found an increased mortality rate in the corticosteroids group and no difference in the incidence of pneumonia [31]. A larger study is merited to study the effect of corticosteroids after trauma.

Difficulties in the supply and availability of blood products with the risk of infections and immunomodulation justify the development of safe and effective hemoglobin-based oxygen carriers (HBOCs). However, the first-generation HBOCs led to systemic and pulmonary hypertension with decreased cardiac output, myocardial damage, and other effects, such as NO scavenging, oxidative stress, and hyperoxia. Second-generation HBOCs are currently undergoing active investigation. These agents seem better tolerated and resulted in fewer complications related to NO depletion. Conjugation of hemoglobin with polyethylene glycol (PEG) is a potentially promising agent. PEGylation increases viscosity, which induces a greater endothelial sheer stress and local NO production with a concomitant increase in functional capillary density [43]. Moreover, PEGylation can increase the oncotic pressure and promote intravascular volume expansion. Two phase III trials have demonstrated that oxygenated PEG-modified hemoglobin (MP4OX) administration was associated with a significant decrease in the incidence of hypotension in patients undergoing primary hip arthroplasty with spinal anesthesia [44,45]. Presently, a study is evaluating the safety and efficacy of MP4OX in trauma patients who suffer from lactic acidosis due to severe hemorrhagic shock. HBOCs could become another tool for clinicians charged with the resuscitation of patients with traumatic hemorrhagic shock.

## Conclusions

Management of trauma patients with hemorrhagic shock is complex and difficult. We recommend managing these patients in centers that treat a high volume of patients (i.e., trauma centers). During recent decades, despite our increasing knowledge of the pathophysiology of hemorrhagic shock in trauma patients, the mortality rate continues to remain high. The role of the physician is to maintain oxygen delivery, despite ongoing bleeding, and to limit tissue hypoxia, inflammation, and organ dysfunction. At the same time, the physician must maintain surgical and arteriographic control of the bleeding and treat coagulopathy to stop hemorrhage in these patients. The optimal resuscitative strategy remains controversial. To move forward, we need to establish optimal therapeutic approaches with clear objectives for fluid resuscitation, blood pressure, and hemoglobin levels to guide resuscitation and limit the risk of fluid overload resuscitation and transfusion.

## Competing interests

Jacques Duranteau has financial competing interests with Laboratoire français du Fractionnement et des Biotechnologies and Fresenius companies.

## Authors’ contributions

AB, AH and JD were responsible for the drafting of the manuscript. All authors read and approved the final manuscript.
